# Contribution of Subway Expansions to Air Quality Improvement and the Corresponding Health Implications in Nanjing, China

**DOI:** 10.3390/ijerph18030969

**Published:** 2021-01-22

**Authors:** Meina Zheng, Xiucheng Guo, Feng Liu, Jiayan Shen

**Affiliations:** 1 Jiangsu Key Laboratory of Urban ITS, Southeast University, Nanjing 210096, China; kumiko1023@163.com (M.Z.); 101002320@seu.edu.cn (X.G.); 230149532@seu.edu.cn (J.S.); 2 Jiangsu Province Collaborative Innovation Center of Modern Urban Traffic Technologies, Southeast University, Nanjing 210096, China; 3 School of Transportation, Southeast University, Nanjing 210096, China; 4 School of Economics and Management, Southeast University, Nanjing 210096, China

**Keywords:** subway, air pollution, difference-in-differences analysis, network density

## Abstract

With China’s rapid economic development, particularly its accelerated urbanization, air pollution has been one of the serious environmental issues across China. Most major cities in China expand their subway systems to handle this problem. This study takes both long- and short-term effects of subway expansions on air quality and its corresponding health implications into account based on a network density-based time series analysis and a distance-based difference-in-differences analysis. The daily and hourly monitor-level air quality data on Nanjing from 13 May 2014 to 31 December 2018, combining with corresponding weather variables, are used to quantify the effect of subway expansions on local air pollution caused by eight new subway lines in Nanjing. The results reveal that subway expansions result in a statistically significant decrease in the air pollution level; specifically, the air pollution level experiences a 3.93% larger reduction in the areas close to subway lines. Heterogeneous analysis of different air pollutants indicates that the air pollution reduction effect of subway expansions is more significant in terms of Particulate Matter (PM_2.5_) and CO. A back-of-the-envelope analysis of the health benefits from this air improvement shows that the total number of yearly averted premature deaths is around 300,214 to 443,498. A set of alternative specifications confirm the robustness of our results. These results provide strong support for putting more emphasis on the environmental effect of subway expansions in the cost-benefit analysis of subway planning.

## 1. Introduction

In recent years, many cities in China have experienced a deteriorating environmental quality, which not only poses a threat to residents’ health and life but also challenges sustainable urban development. Zhao et al. [1] found that anthropogenic PM_2.5_ (fine particulate matter with a diameter of 2.5 micrometers or less) exposure in China resulted in 1.08 million premature deaths in 2012. In addition to PM_2.5_, other types of air pollutants like nitrogen oxide (NOx), sulfur dioxide (SO_2_), carbon monoxide (CO), and photochemical smog and their derivatives through physical and chemical reactions also induce serious health problems in China. A large body of literature has confirmed the link between vehicular emissions and air pollution [2,3] and the corresponding health problems [4]. For instance, Li and Yin [5] calculated the share of traffic-related air pollution in urban total air pollution emission and found that 63% of CO and 37% of NOx were generated by urban vehicular emissions in China. With the increase of private vehicles in China, traffic-related air pollution may become more and more serious if there is no adoption of appropriate environmental policies to improve traffic-related air quality. Given the advantage of public transit, such as buses, subways, and other types of rail transit, in reducing traffic congestion and air pollution [6,7], many cities in China joined the “rail transit club” to counter urban air pollution. By the end of 2019, 40 cities opened rail transit, reaching a total of 6736.2 km, of which 5180.6 is in the form of subway lines. From 2013 to 2019, Chinese annual average investment in transportation infrastructure stands at more than two trillion Yuan. Although urban planners and policymakers have realized the importance of rail transit in improving air quality and traffic conditions, scholars and urbanites still doubt the actual effect of it. First, the positive connection between the expanded subway and decreasing local pollution is still unclear in China. Second, the construction and operation costs may exceed the benefit that comes from it. Therefore, a precise identification and measurement of the transport and air quality effect from expanded subway play a vertical role in the assessment of new subway construction.

Previous works on the relationship between urban subway and air quality suggest two aspects. First, the substitution effect, that is enhanced subway will motivate more commuters to transfer from cars to subways, curbing vehicular emissions [8]. In general, the negative externality of rail transit like noise pollution, traffic accidents, and air pollution is far less than that caused by private vehicles. Zheng and Kahn [9] found that the costs of accidents per person kilometer and the emissions of CO_2_ of rail transit are only 5 percent and 4 percent of private vehicles’ respectively, based on the analysis of European cities. This improvement mainly results from the substitution of previous energy-intensive or highly polluting travel mode to low-carbon trip mode. Since marginal drivers who switch to public transport tend to be poorer among all drivers, who may drive particularly dirty cars, thus the substitution effect could function through affecting the composition of the stock of vehicles on the road [7]. In this connection, improved rail services, particularly the increase in frequency, will contribute to reducing air pollution concentration [10]. Based on the satellite imaging and a cross-country comparison in terms of subway expansions from 2000 to 2014, Gendron-Carrier et al. [7] found a 4% reduction in particulate concentrations. Through the natural experiment of a public transit strike in the five largest German cities, Bauernschuster et al. [11] found a statistically significant increase in particulate pollution (PM_10_) during this strike. Moreover, the improved subway is also capable of reducing traffic congestion and improving commuters’ accessibility [12], and then vehicle emissions will be curbed during this process.

Another stream of research on this topic puts more emphasis on the creation effect, that is improved transit may encourage some latent drivers to transfer from buses or subway to cars. Meanwhile, more residents are likely to move to the suburban area, which results in more commuting demand and a longer daily commute, ultimately deteriorating air quality and exacerbating traffic congestion [13]. Based on a panel of 96 urban areas across the U.S. from 1991 to 2011, Beaudoin and Lawell [14] did not confirm the positive association between public transit expansion and the improvement in urban air quality. This is different from Bauernschuster et al.’s research, that confirmed the positive connection between the misfunction of public transit and the deterioration in urban air quality [11]. Rivers et al. [15] found a short-run decrease in ambient NOx concentrations when public transportation was out of service due to transit union strikes in 18 Canadian cities. Therefore, there is still no consensus on the effect of subway expansions on local air quality.

Concerning China, most studies highlight the substitution effect of rail transit. Chen and Whalley [6] examined the change of central Taipei’s air pollution before and after the operation of the Taipei subway through a regression discontinuity analysis and found a 5–15% reduction in CO. Similarly, Zheng et al. [16] employed the difference-in-differences (DID) approach to examine the medium-term change in the air pollution level of Changsha, China, out of the opening of its first subway line and found an 18.1 percent larger reduction in CO in the vicinity of new subway stations. Li et al. [17] also used a DID method to examine the impacts of new subway lines in Beijing on local air quality from 2008 to 2016 and found a significant improvement in air quality. In addition to the analysis aimed at one single city, researches on a cross-city comparison in terms of the relationship between the expanded subway and local air quality also suggest the existence of the substitution effect. Based on 45 newly opened rail lines of 14 cities in China, Liang and Xi [18] found that the improved rail transit may induce more commuters to escape from cars, especially taxis, which contributes to the improvement in air quality. Gu et al. [19] examined the relationship between transit-oriented development and air quality in 37 Chinese cities, and also found that rail-based transit-oriented development contributes to better air quality. However, some researchers also found the insignificance of the substitution effect in China. Zhang et al. [20] found that improved rail transit has a small and statistically insignificant effect on the travel mode of residents with cars after the analysis of a survey data on travel energy consumption in Beijing. Wang et al. [21] found that the short-term impact of subway expansion on PM_10_ tends to be positive, while negative in the longer term based on the PSM-DID method.

In summary, the transport−air pollution literature predicts that the final impact of subway expansions on air quality could go in either direction. Although some researchers have examined this relationship in the context of China, most of them choose Chinese megacities like Beijing and Shanghai as the target of interest [22,23], with little focus on medium-sized cities. What is the specific effect of subway expansions on local air quality, particularly in the context of Chinese medium-sized cities? Is there significant heterogeneity among different types of air pollutants in terms of the degree of influence? How to deal with the endogeneity concern? The dearth of attention to these questions informs us that a more integrative understanding of subway development and environmental pollution is indispensable.

Therefore, this paper presents a case study of the impact of subway expansion on air quality in Nanjing and based on a distance-based DID approach used to solve the endogeneity concern. Its objectives are to (1) identify the relationship between subway construction and local air pollution, and (2) achieve a comprehensive understanding of the air pollution reduction mechanism of urban rail transit through considering the heterogeneous effect of different types of air pollutants and different time intervals based on hourly air pollution data. Our study may contribute to the transportation infrastructure–air quality literature in the following four areas. First, this study adopts high-frequency air quality data at the monitor level together with subway lines in the same city to identify the causal relationship between subway expansions and air pollution. Second, we construct a continuous subway density indicator to reflect the subway network density change, which allows us to identify subway expansions’ marginal impact instead of only focusing on the magnitude of air quality change due to the first subway line. Third, a distance-based DID approach that considers the endogeneity in subway density is adopted to handle this concern. Last, heterogeneity tests for different types of air pollutants and different time intervals within a day are performed using hourly air pollutant emission data.

The remainder of this paper is structured as follows. In the second section, we discuss the empirical background in the context of Nanjing, including the situation of air pollution and subway development in Nanjing. Section 3 describes the key data, followed by the empirical strategy. Section 4 presents the main empirical results, and then, on this basis, Section 5 presents a back-of-the-envelope analysis of the health benefits. Section 6 proposes corresponding policy suggestions and Section 7 concludes. Last, Section 8 presents a brief discussion of future research on this topic.

## 2. Background on Air Pollution and the Subway System in Nanjing

### 2.1. Air Pollution in Nanjing

**Figure 1:**
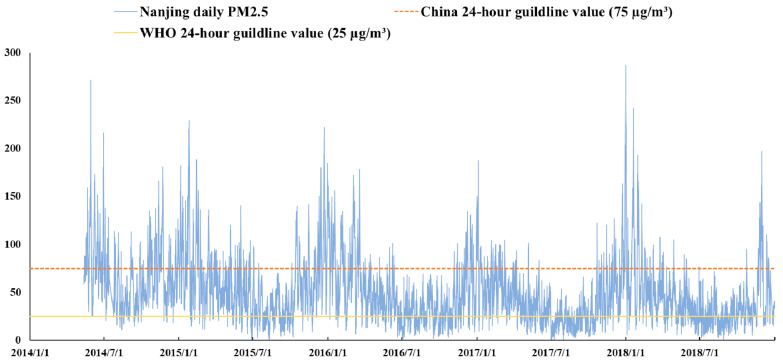
The daily change of Nanjing Particulate Matter (PM_2.5_) concentration (µg/m^3^). Notes: Data come from 9 monitors within Nanjing, as these monitors had been established before 2014, their number and locations do not change over time.

Over the past decades, sustained and rapid economic growth has been accompanied by an increasingly deteriorated environment in China. Nanjing, as the capital of Jiangsu Province, is a highly urbanized and industrialized city as well as the central city in the northwestern Yangtze River Delta (YRD). Nanjing also experiences a gradually deteriorating air quality along with its rapid economic development. Figure 1 shows the daily change in PM_2.5_ concentrations in Nanjing from 13 May 2014 to 31 December 2018. The concentration level of most days during this process is above the World Health Organization (WHO) 24 h guideline value, and the yearly average level is up to 52.4 µg/m^3^, that is about two-thirds of the Chinese annual standard.

Air pollution represents the largest single environmental health risk over the world according to the WHO [24]. A 2013 assessment by the International Agency for Research on Cancer (IARC) of WHO also concluded that the association between ambient air pollution and an increase in cancer incidence had been confirmed, especially lung cancer and cancer of the urinary tract/bladder (See: https://www.who.int/news-room/fact-sheets/detail/ambient-(outdoor)-air-quality-and-health for more information). Power plants, vehicles, and industrial activities all contribute to the increase of outdoor air pollution, though identifying the specific share of transport-related sources is challenging as vehicular emissions usually induce secondary air pollutants after physical or chemical processes. However, according to the statistics of China’s National Bureau of Statistics (CNBS), the annual average concentration of PM_2.5_ in Nanjing was 74 in 2014, while only 43 in 2018. Its subway system, meanwhile, has achieved a well-developed transport network and the number of trips by public transport have accounted for 50% of total travel in the urban area. Whether the link between both changes exists need further empirical evidence.

### 2.2. Subway System of Nanjing

**Figure 2:**
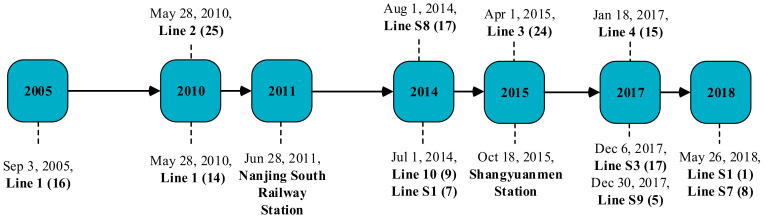
The timeline of Nanjing’s subway development. Notes: The number in parentheses denotes the number of stations. Nanjing South Railway Station opened accompanied by the opening of Beijing–Shanghai high-speed railway (HSR) in 2011, which has been the most important traffic hub in Nanjing. The opening of Shangyuanmen Station in Line 3 was delayed due to the engineering construction.

Before 2014, Nanjing only had two subway lines in operation, with 55 stations, and the time gap between the first subway line and the second one was five years (Figure 2), which suggests a slow stage of development. Through hosting the second Youth Olympic Games (YOG) in 2014, Nanjing entered the era of acceleration in subway construction. From 2014 to 2018, 8 new subway lines were constructed, reaching a total of 378.38 km. This operating kilometrage makes Nanjing rank No. 4 among China’s 37 cities with the subway, and also the first city in China that achieved the full coverage of access to subway services across all districts.

**Figure 3:**
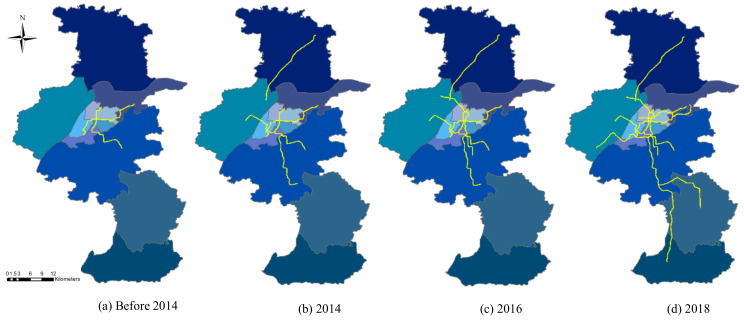
The annual change in the subway network of Nanjing. Notes: (**a**–**d**) map the subway network of Nanjing at the end of 2013, 2014, 2016, 2018, respectively.

Figure 3 shows the detailed change in the subway expansion of Nanjing from 2014 to 2018, where a well-developed subway network has been gradually achieved. Another 7 subway lines are currently under construction, due to be completed in 2022. There will be 25 subway lines, and the total length of operational subway lines will be extended to 1011.2 km by the end of 2030, according to Nanjing City Urban Master Plan (2007–2020).

## 3. Data and Methodology

### 3.1. Data Sources and Summary Statistics

**Figure 4:**
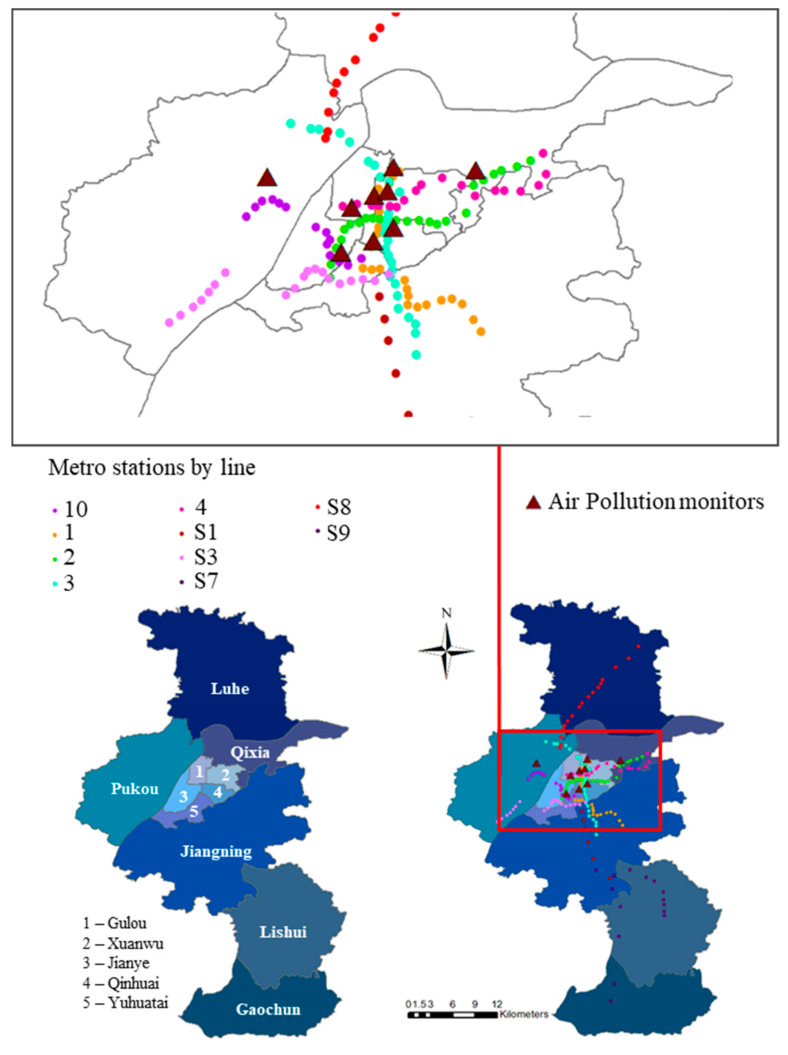
The spatial distribution of the nine air quality monitors and subway stations in Nanjing.

Different from previous research based on the city-level air pollution data, this study collects Nanjing’s air quality data at the monitoring station level (the geographic distribution of 9 monitors is shown in Figure 4). Due to the limit of data availability, we collected the daily and hourly monitor-level Air Quality Index (AQI), SO_2_, NO_2_, CO, PM_10_, PM_2.5_, and Ozone (O_3_) from 13 May 2014 to 31 December 2018. During this period, 8 new subway lines with 104 stations were constructed, accounting for almost two-thirds of the total in operation by the end of 2018. The AQI is based on the abovementioned six atmospheric pollutants that measure daily air quality according to a scale of 0 to 500: the higher the value, the greater the air pollution and health risks.

The main explanatory variable in this study is the indicator that reflects the opening dates and locations of subway lines. Figure 4 shows the layout of monitoring stations and subway stations in Nanjing in 2018, most of the high-supply areas of subway services lie in the urban area around the city center. Considering that almost all stations in the same line opened on the same day, we chose seven major opening dates during the sample period (as shown in Figure 3) as the date of interest.

**Figure 5:**
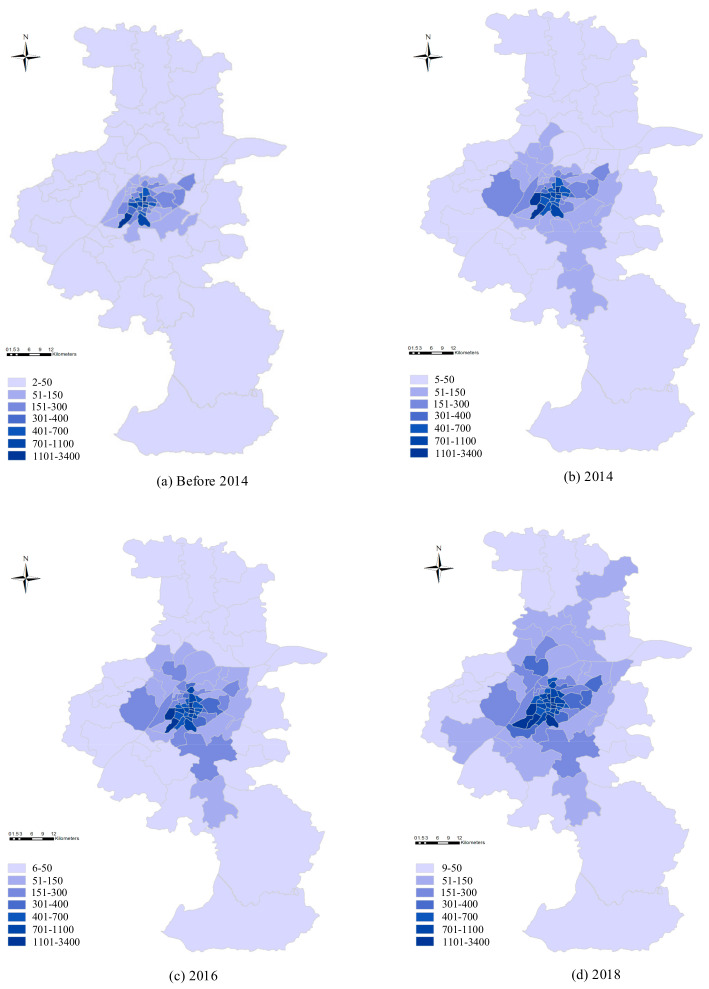
Subway expansion and network density at the block level. (**a**–**d**) show the subway network density at the block level at the end of 2013, 2014, 2016, and 2018, respectively.

In addition, we also constructed a continuous variable to reflect the subway density:

(1) $$Subway\_density_{it}=\underset{j\in N_{t}}{\sum }\frac{1}{Dist_{ij}^{2}}$$

where i, j, and t represent air quality monitors, subway stations, and days, respectively. $N_{t}$ indicates the number of currently operational subway stations at time t. $Dist_{ij}^{2}$ indicates the square of the distance from monitor i to subway station j at time t. This measure could be regarded as a transformation of the gravity model in physics, which reflects the number of subway stations centered around a specific monitoring station. More subway lines indicate higher subway density, however, monitors that are closer to new subway lines will have a higher level of density. Therefore, these stations are expected to record a better air quality as commuters nearby are more likely to substitute from cars to the subway. Figure 5 reports the subway density changes of each block (calculated based on Equation (1)) across the city with the subway expansion. In general, the subway density of each block decreases with the increase of distance between the block of interest and urban center. Both air quality and subway datasets are aggregated to the monitor and daily level.

We also included daily weather variables, including daily maximum and minimum temperature, average wind speed, and binary variables, indicating rain or snow and the same wind direction from day to night, as local weather conditions are important determinants of air quality [7]. First, sun and high temperatures can function as catalysts for chemical processes of air pollutants. Second, air pollutants will be removed from the atmosphere by precipitation. In view of the fact that the unit of analysis is based on daily data, we also added a set of time-fixed effects (year, month, weekend, season, and holidays). The meteorological data come from the China Meteorological Administration (CMA). The industrial scale and structure, investment in infrastructure, vehicle fleet, and other socioeconomic factors are not controlled due to their unavailability at monthly or seasonal level. Given this potential problem of missing variables, we adopted a DID method with a 60-day time window around the opening dates of new subway lines to handle it, and we assume that the erratic fluctuation of economic activities will not appear in the short term. Table 1 summarizes the descriptive statistics for the main variables of our analysis.

### 3.2. Econometric Model

In this section, we first employ subway network density as the key independent variable to examine the effect of subway expansions on urban air pollution. This measure relies on the spatial and temporal variation of the network expansion during the data period that reflects a long-term change of the subway network. Then, we use the DID method, focusing on a shorter time window, as an alternative strategy to confirm the robustness of our result and investigate the heterogeneous effects of different air pollutants. All regressions are performed through the software of Stata 16.

#### 3.2.1. The Relationship between Subway Density and Air Quality

In our primary econometric exercise, we examined changes in air pollution around the air quality monitors and estimated the conditional correlation between it and subway density in Nanjing over time. We specify a reduced form model to quantify the effects of a marginal increase in subway supply on equilibrium air quality in the vicinity of a monitor. For each pollutant, p∈{AQI, PM_2.5_, PM_10_, CO, NO_2_, SO_2_, O_3_}, recorded by monitor i at time t:

(2) $$ln\left(Air\_pollution_{pit}\right)=\theta_{1}Subway\_densityst_{it}+\theta_{2}Monitor_{i}+\theta_{3}Trend_{it}+\theta_{4}Weather_{t}+\theta_{5}\mathrm{Time}\;\mathrm{fixed}\;\mathrm{effect}+\delta_{it}$$

where $ln\left(Air\_pollution_{pit}\right)$, the dependent variable, is the daily air pollution level recorded by each monitor, and each pollutant data is logarithmically transformed to avoid nonnormality and heteroscedasticity. $Monitor_{i}$ indicates the monitor fixed effects to control unobserved location attributes that may affect air quality, these attributes do not change over time but change across monitors. $Trend_{it}$ is a monitor-by-day variable (i.e., the interaction of the dummy for monitor i and the linear time trend t) that picks up time-varying monitor-specific trends and alleviates the endogeneity concerns in terms of the location of subway lines as well as helps to solve the spurious regression problem. $Weather_{t}$ is a vector of weather covariates including dummies for daily snow and rain (rain or snow = 1, otherwise = 0) and fixed wind direction (i.e., the corresponding dummy will be set as 1 if the wind direction of day and night keeps consistent, otherwise 0), wind speed, as well as daily maximum and minimum temperature. Time fixed effect controls a set of temporal fixed effects to capture those time-varying unobservables, including year, season, weekend (weekend = 1, weekday = 0), and holiday fixed effects (holiday = 1, otherwise = 0). $\delta_{it}$ is an error term.

The empirical approach above focuses on the subway density change and its impact on the air quality, while the measurement is at the station level, not a city-wide level. Therefore, we expect that there exist significant differences in this impact between monitoring stations with denser subway networks nearby and those who are far away from subway lines.

#### 3.2.2. Difference-in-Difference Specification

A simple time-series regression on the casual relationship between subway density and air pollution has the potential problem of endogeneity. That is, the locations of new subway lines are not placed randomly but are strongly correlated with active economic activities, which usually inform worse traffic congestion nearby. This non-random placement may lead to the downward bias of the estimated coefficient of subway density in Equation (1). Moreover, those cities with subway lines are usually accompanied by serious traffic congestion and air pollution, and the general Ordinary Least Square method (OLS) may induce reverse causality and self-selection bias [25]. The omitted variable bias may also result in endogeneity, which weakens the robustness of estimating results [26,27]. To address concerns of endogeneity, we also estimate a DID specification. Specifically, we use the OLS method to estimate the following DID model:

(3) $$ln\left(Air_{p}ollution_{pit}\right)=\beta_{1}Subwayn_{it}\times WPost_{t}+\beta_{2}Monitor_{i}+\beta_{3}Trend_{it}+\beta_{4}Weather_{t}+\beta_{5}\mathrm{Time}\;\mathrm{fixed}\;\mathrm{effect}+\delta_{it}$$

where $Subwayn_{it}$ is an indicator variable that takes a value of one for monitor i if it is within 2 km of any operational subway stations that were opened on a given date, od (od–60≤t≤od+60), and a value of zero for others. $Post_{t}$ is a dummy variable that takes a value of one for all 60 days after these new subway stations are operational (od≤t≤od+60), and a value of zero otherwise. W indicates the corresponding unit vector. After interacting $Post_{t}$ with the corresponding unit vector W, one will get a vector of treatment indicator for each monitor on each date. Our coefficient of interest, $\beta_{1}$, will measure the change in air pollution due to subway opening for areas near new subway stations over the 60-day period following the opening date. The rest of the control variables are the same as Equation (2). We set 2 km as the cut-off value according to the research of Li et al. [17], where they assume that the typical walking and biking distance is 1 and 3 km, respectively. A mean value of them is taken as the radius that a subway station can exert impact on commuters’ choice of travel mode. The 60-day windows on either side of the 7 opening dates of new subway lines allow us to examine the impact of each new subway line on the air quality separately and avoid the overlap of each new line’s time window.

Before the analysis with the DID approach, a test of parallel trends in pre-treatment periods should be conducted. In this study, the air quality in treated and untreated subclasses should follow similar trends before the subway opening date. We decompose the study window into 20-day bins and estimate the following set of regressions:

(4) $$ln\left(Air\_pollution_{pit}\right)=\underset{m\in -2,\ldots ,3}{\sum }\partial_{m}F_{t}\left(m\right)Subwayn_{it}+\beta_{2}Monitor_{i}+\beta_{3}Trend_{it}+\beta_{4}Weather_{t}+\beta_{5}\mathrm{Time}\;\mathrm{fixed}\;\mathrm{effect}+\delta_{it}$$

where $F_{t}\left(m\right)=W\left[\mathrm{od}+20\times \left(m-1\right)\leq t\leq od+20\times m\right]$, and we set the 20-day windows before the new subway lines’ opening dates (i.e., m=0) as the base interval. The coefficient $\partial_{m}$ captures the differences in trend between observations of pre-openings and post-openings. The results are shown in Table 2.

It could be found that there exist no significant changes in air quality between the treated and untreated subclasses in all pre-opening intervals compared with the base interval. In contrast, we find a significant air pollution improvement effect in all three post-opening intervals in the specification that does not control the monitor-specific time trend. Therefore, the treatment and control group satisfy the parallel trends assumption for DID analysis.

## 4. Empirical Results

### 4.1. Estimates Based on the Subway Network Density

Table 3 presents the results from OLS estimates based on Equation (2) (the original regression data and the corresponding estimation codes are available upon request). This analysis seeks to estimate the conditional correlation between subway density and air quality, where columns (1), (2), and (3) report the results of AQI as the dependent variable. We only control weather variables and time-fixed effects in column (1), and the coefficient suggests a positive connection between subway density and air pollution. The main reason may be that areas with a denser network are usually located in the city center, where more pollutants tend to concentrate. After adding the monitor fixed effect in columns (2) and (3), this positive correlation disappears. Considering the endogeneity of subway line location (i.e., subway locations are usually closely related to economic activities), column (3) adds monitor-specific time trends to the specification. The downward bias may be generated in the absence of this control variable, and the difference of estimated results in columns (1) and (2) has confirmed it. We see that the magnitude of subways on air quality, $\theta_{1}$, is about −0.071 and is different from zero at the 1% level after controlling the monitor-specific trends. That is, a one standard deviation increase in subway network density reduces the air pollution level by about 7.1 percent, and this result is statistically significant at the 1% level.

Estimated results concerning other air pollutants in Table 3 suggest a similar relationship, except for the O_3_. This positive effect on O_3_ pollution is possibly related to the generation process of it. O_3_ is the reaction product of NOx and volatile organic compounds (VOCs) catalyzed by high temperatures and ultraviolet light [28]. It is more related to human activities that are positively associated with the density of subway stations. All the estimations of weather variables are consistent with our intuitive judgments. High temperature contributes to the increase of air pollution level, while high wind, constant wind direction, low temperature, and rain or snow all help the dispersion of air pollutants.

### 4.2. Additional Evidence Based on a Difference-in-Differences Analysis

Considering the potential endogeneity concern of the estimation of time-series correlation above, in this section, we use a DID method to confirm the robustness of our results. Table 4 shows the estimation result using the DID method, specified in Equation (3). We sequentially added weather variables, time, and monitor fixed effects as control variables from column (1) to (5), and all report similar results with the results in Table 3. That is, there exists a negative correlation between subway openings and air pollution levels. Specifically, column (5) suggests that a subway opening reduces air pollution level by 3.93 percent for monitors near new stations (≤2 km) compared with those far away from these stations. We also chose 4 km ($Subwayn4_{it}$) as the cut-off radius that distinguishes the treatment group from the control group. The estimation coefficients are reported in column (6) in Table 4. The results change very little compared to the results in Table 3, which confirms the robustness of our estimation. We also collected the hourly air quality data in the same sample period, and the results are reported in column (7) in Table 4, which are similar in sign and magnitude to those in Table 3.

The regression results of DID above focus on average effects over the 60 days before and after a subway opening, while different time windows may have a certain impact on the estimation results. Therefore, we used 10, 20, 30, 40, 50, 70, 80, 90, 100, 110, 120, 130, 140, 150, 160, 170, and 180-day windows as alternative specifications, and the results are reported in Table 5. These estimates are broadly consistent with the inspection of Table 4: subway expansions have a negative effect on air pollution levels during each 40 or longer day period following a subway opening. In the short term, commuters may need some time to switch to rail transit, which results in a positive subway effect on air pollution. When extending the time window to 40 days, the subway effect on air pollution starts to be negative; however, this effect fades away during the 120-day and longer post-opening. With the improvement of Nanjing’s subway, the daily average passenger volume by subway had reached 1.47 million in 2014 and 3.07 million in 2018, which has more than doubled within four years. While, during the 4 years (2014–2018), Nanjing incremental private vehicles were 586,890, reached 2.07 million in 2018, and the population experienced slow growth during the 4-year period. This implies that the reincorporation of these latent drivers is likely to counteract the substitution of auto trips for public transit trips and improvements in air quality. Therefore, we infer that improved transit due to the subway expansion may induce some latent drivers to travel by cars, which weakens the subway effect on air pollution reduction in the long term. In addition, considering that people’s choice of travel mode may be endogenously related to air quality, for instance, people tend to travel by subway during the “red alert” days, we omit the samples with good (AQI ≤ 50) or poor (AQI > 300) air quality, and the estimation result is reported in column (18) of Table 5. Results are also very similar to those in Table 3.

### 4.3. Heterogeneous Effect

Regression results so far focus on daily average air quality, and intra-day heterogeneity has not been considered. High levels of tailpipe emissions usually appear at peak travel times, while the subway is out of service between 11:00 pm and 5:00 am on any given day. Whether there are distinct patterns for the subway effect on air pollution at different intervals within a day needs further investigation.

**Figure 6:**
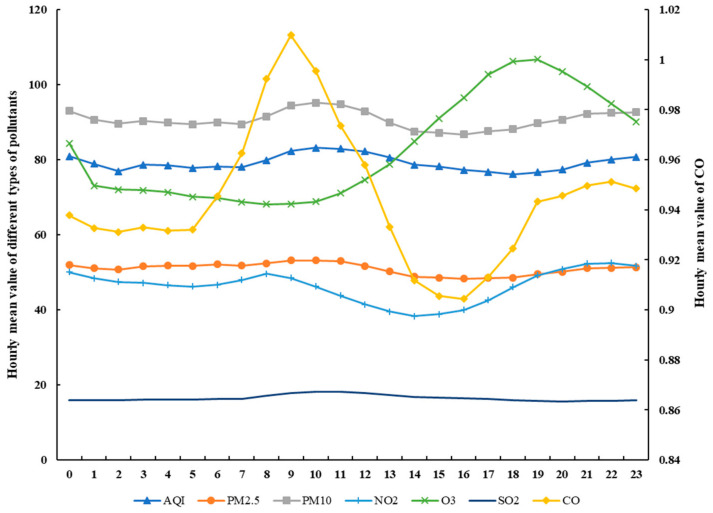
The average level of air pollution in each hour within a day.

Before the empirical examination, we plot the change in hourly pollutant concentration in Figure 6. We can find that AQI shows a relatively stable trend within a day: a slow upward trend from 6:00 to 12:00 am may result from the increase of CO, PM_10_, and PM_2.5_ concentrations in the same period. Moreover, the CO concentration have shown a volatile trend, and its daily maximum pollution level typically occurs at about 9:00 am, which synchronizes with rush hour periods. This trend reflects the contribution of tailpipe emissions to the deterioration of air quality. For the NO_2_, we can see a downward trend from 8:00 am to 3:00 pm, and an upward trend from 4:00 pm to 2:00 am. As the NO_2_ concentration is closely associated with vehicle emissions and industrial production activities, its variation keeps pace with commuting and factory emissions at night. PM_10_ and PM_2.5_ show a similar trend within a day, that is a slow increase during the 6:00 to 10:00 am and 4:00 to 11:00 pm stretch, which suggests their response to the morning and afternoon travel peak. The minimum PM_2.5_ and NO_2_ recorded in the afternoon may result from the comprehensive effect of photosynthesis of plants, meteorological factors, and commuting time. The O_3_ experiences a down and up changing trend, and the peak values appear at about 7:00 pm—this is more likely related to its generation process that we discussed above [28]. As the main contributor to SO_2_ is industrial emission, it will not show any time-varying characteristics; hence, SO_2_ is the least volatile air pollutant.

Based on the descriptive analysis above, we estimate Equation (3) for three different time intervals in a day (i.e., subway service time: 6:00 am to 11:00 pm, travel peak time: 7:00 to 9:00 am and 5:00 to 7:00 pm, and subway out of service time: 1:00 to 5:00 am). The results are reported in Table 6, from where we can find that the negative subway effect on air pollution still works, except for NO_2_ and O_3_. The subway opening causes a statistically significant reduction in PM_2.5_ and CO concentration, which are also the major components of vehicle exhausts. This finding is consistent with Wei’s [29] estimation results using panel data of 16 major cities in China from 2014 to 2017. This reduction effect is significant both during subway service time and travel peak time, informing us that subway expansion contributes to the reduction in air pollution level.

The main source of NO_2_ is vehicle emissions, and the concentration of it is particularly localized near major roadways [30], where the monitors of the treatment group in this study are also located. Commuters’ demand for subway is higher during rush hours when pollutant concentrations are also elevated, especially near those major roadways. In contrast, inside of the 1:00 to 5:00 am stretch, the parameters of NO_2_ and PM are statistically insignificant. In addition to the subway, demand for buses is also higher during rush hour periods accompanied by high congestion, and buses generally emit pollutants at a higher rate than auto travel on a per vehicle-mile basis [14,15]. Therefore, air pollution levels may be higher within a day during the service time, which may be captured by the DID approach. Our DID analysis focuses on the subway effect on air quality in the short-term based on the hourly concentration data, which may capture a distinct pattern from the method based on the whole data period. Columns (2), (8), (14), and (20) of Table 6 show that subway expansion is associated with a statistically insignificant decrease of PM_10_ concentrations, which further confirms this assumption. In terms of the O_3_, the positive coefficient is related to its generation process that has been described above. The insignificant coefficient of SO_2_ also confirms the findings in the descriptive analysis above. One concern with our research design may be that the subway opening is picking up other systematic unobservable variables. We should not see a significant reduction effect when the subway is out of service, and we probed this claim via a placebo test. Columns (13) to (18) show the subway effect on air quality from 1:00 to 5:00 am in a day. Except for CO and O_3_, other coefficients became statistically insignificant, and this is consistent with our main model. Columns (19) to (24) report the results based on hourly data of a whole day.

## 5. Health Implication of Subway Expansions

This section presents a back-of-the-envelope analysis of the health benefits from air improvement attributable to subway expansions. We refer to the research of He et al. [31] to predict the reduced mortality out of the air quality improvement:

(5) $$Mortality_{i}=\Delta AQL_{i}\times Elasticity\times BaseMR_{i}\times Popu_{i}$$

where $Mortality_{i}$ indicates the estimated prevented deaths of city i during the sample period. $\Delta AQL_{i}$ represents the estimated change in air quality level in city i during the sample period, where we calculate it based on Equation (3) with $\ln\left(PM_{2.5}\right)$ as the dependent variable. Elasticity represents the sensitivity of mortality to a one-unit change in air quality. Since its estimate is not the focus of interest in this study, we use the estimates from existing researches on the effect of air pollution on human health. Since the impacts of air pollution on human health vary over time, researches in this field based on data from earlier years may be less effective as the reference. Moreover, considering the credit of estimates based on quasi-experimental studies compared with those based on associated regression models [32], we used Web of Science, Google Scholar, Scopus, and other databases to identify academic articles and book chapters that meet these criteria, and it could be found that eligible articles in this field are not that rich. Finally, Fan et al. [33] found that a 10 µg/m^3^ increase in PM_2.5_ resulted in a 2.2 percent increase in mortality rate based on a Regression Discontinuity (RD) analysis, and He et al. [34] proposed that this rate can reach over 3.25 percent through an estimation of the effect of straw burning on air pollution and health in China, and these were chosen as two main references. Therefore, we set the range of mortality increase as 2.2~3.25% following a 10 µg/m^3^ increase in PM_2.5_. $\mathrm{Base}MR_{i}$ denotes the annual mortality rate in city i at the base year, and $Popu_{i}$ indicates the population in city i at the base year. In view of the data availability, we set 2018 as the base year of mortality rate and population. If we assume that Nanjing is a representative city in China, the same air improvement could be achieved through more public transport supplies in other cities of China. The results show that the total number of yearly averted premature deaths is around 300,214 to 443,498 due to the air quality improvement caused by the development of public transport infrastructures. This reflects the enormous social costs of air pollution during the normal time.

## 6. Policy Implications

Understanding how subway expansion affects air quality based on the high-frequency air pollution data is essential for crafting efficient environmental policies to alleviate the negative effects that air pollution brings on public health and welfare from the perspective of public transportation. In this connection, we propose the following policy recommendations to deal with the public transport–air pollution nexus and take full advantage of public transport’s potential in improving urban air quality.

First, planners and policymakers should take more integrated measures that consider the cost of subway construction and the social value of it, especially taking the environmental effects into account simultaneously when conducting a cost-benefit assessment of new subway lines [6].

Second, the government should tighten tailpipe emissions standards and encourage lower emission vehicles. Meanwhile, attention should also be paid to vehicle-related supporting infrastructures, particularly the expansion of the road surface area for public transport and bicycle lanes. Both measures will contribute to solving urban traffic congestion and developing a fast, safe, and convenient transportation condition. However, the one-size-fits-all approach, such as the driving restriction policy and the license-plate lottery policy, should be avoided during this process. Otherwise, these executive orders may distort consumers’ behaviors of purchasing cars and lead to a skewed distribution of high-polluting vehicles [35] and restrain residents’ travel demand, hence resulting in a decrease in social welfare [36]. Moreover, wider road surface area for bicycle lanes encourages daily exercise by cycling from home to school or work or by walking to and between stations of public transportation, all of which contribute to reducing urban traffic and air pollution as well as improving individual health. In addition to the expansion of the road surface area, providing changes to public traffic in the periphery of Nanjing by park and ride facilities with charging stations for electric cars and serving larger areas with attractive parking places for bikes at metro stations can be considered to fully unlock the subway’s potential to reduce pollution, as these measures could also reduce commuting by car to and from suburbs and surrounding areas, and thereby reduce air pollution.

Third, other modes of public transport, such as shared bikes, should be encouraged to connect subway stations and destinations, ultimately forming a synthesis transportation system. The shared bike, as an easy and low-cost mode of transportation, contributes to the reduction in air pollution, noise, and traffic congestion and has been a popular travel mode in China since it first entered the public arena in late 2016 [37]. Although some management problems, such as disorder of bicycle parking and the misfunction of the deposit–refund system, have arisen in the development of the market-oriented shared bike, the average number of commuters reached more than 40 million a day by 2019 [38]. Therefore, there is much room left for improvement to drive the rapid and healthy development of the green travel mode.

Last, policymakers should improve public awareness of the effectiveness and benefits of public transport in reducing air pollution, and help residents form the habit of green travel. It is worthy of note that this transition cannot happen overnight; thus, it will require patient guidance and encouragement along the way.

## 7. Conclusions

Given the deteriorating air quality across cities in China, central and local governments have taken the improvement in transportation infrastructures as an effective measure to counter it. However, it is hard to know the specific effectiveness or benefits of this measure without the information regarding the magnitude of air quality improvement caused by public transportation. Previous research in this area, in general, focuses more on the congestion relief function of public transport [11,12,39], and less research has been devoted to understanding the subway effect on air pollution.

This paper took both short-term and long-term effects of subway expansion on local air quality into consideration based on a network density-based time series analysis, and a distance-based DID approach with a 60-day window. Different from previous research of similar topics which mainly focused on first-tier cities in China [40,41], this study chose Nanjing, a medium-sized city, as our study area, which allowed us to have a comprehensive understanding of the pollution abatement effect of subway expansions. To shed light on how subway expansion is affecting local air quality, we used daily and hourly monitor-level air quality data on Nanjing from 13 May 2014 to 31 December 2018, combining with corresponding weather variables. We examined the change in air pollution concentration caused by the 8 new subway lines during the sample period in Nanjing. The results showed that there existed a positive effect of subway expansion on local air quality, specifically air pollution level experienced a 3.93% larger reduction in the areas close to subway lines. This decline is similar to the result of Gendron-Carrier et al. [7] based on the analysis of 39 cities across the world with aerosol optical depth (AOD) from satellites as the air quality indicator. However, this effect was inconsistent among different types of air pollutants: the pollution abatement effect is more significant in terms of PM_2.5_ and CO. Chen and Whalley’s [6] analysis with Taipei as the case study also found a significant reduction in carbon monoxide due to the opening of the Taipei Metro. Liang et al.’s [18] research based on 14 Chinese cities also confirmed the significant reduction in CO and particulate concentrations following a subway system opening. A back-of-the-envelope analysis of the health benefits from this air improvement showed that the total number of yearly averted premature deaths is around 300,214 to 443,498. The reduced mortality is larger than that in Liang et al.’s [18] work, who set the environmental effects of the subway in Beijing as the benchmark when calculating the potential health benefits.

## 8. Future Research and Expectations

This study confirms the environmental effects of subway expansions across urban areas in China. However, our analysis focused on Nanjing only, and since the precise effects of subway expansions depend on many factors, which may differ across areas, future research could replicate these results in other contexts and unpack the channel through which subways affect air pollution, and more city-specific policy recommendations could then be proposed. Since the data period in this study is relatively short, future research could further extend the data period and examine the environmental effect of subway expansion within a longer time period. The non-linear effect of subway expansion on air quality may occur in the long run, so the corresponding analysis of non-linear models could be considered. Besides, future research could also examine the impact of subway expansion on health outcomes directly to analyze the role of underlying health conditions in the choice of travel mode. Obtaining reliable and high-frequency data on mortality and the impact of exposure to air pollution on mortality in different cities, combining with the unique traffic patterns, geography, and economic structures of them, would contribute to a more credible estimation of the health benefits of subway expansion in future research.

## Figures and Tables

**Table 1:** Definition and summary statistics of the main variables involved in this study.

Variable	Definition	Obs.	Mean	SD	Min	Max
$AQI_{it}$	Daily air quality index	15,070	78.959	40.315	11.571	337.042
$Subway\_densityst_{it}$	The standardized density centered at a given monitor	15,080	1.996	0.952	0.098	3.155
$rain_{t}$	Rain or snow dummy (1 = there was rain or snow, 0 = otherwise)	15,080	0.353	0.478	0	1
$toptem_{t}$	Daily maximum temperature (°C)	15,080	21.353	9.110	−5.000	40.000
$minitem_{t}$	Daily minimum temperature (°C)	15,080	13.598	9.207	−9.000	30.000
$windd_{t}$	Same wind dummy (1 = same wind direction from day to night, 0 = otherwise)	15,080	0.603	0.489	0	1
$winds_{t}$	Average wind speed (km/h)	15,080	3.609	0.565	1.500	7.500

Notes: i = 1, …, 9 denotes an air quality monitor, t denotes a specific day from 13 May 2014 to 31 December 2018. The standardized density in the vicinity of a given monitor refers to the $Subway\_density_{it}$ calculated from Equation (1) divided by its standard deviation (SD). The unit of observation is monitor-day.

**Table 2:** Parallel trend test.

	$\mathrm{Dependent}\;\mathrm{Variable}:\;ln(AQI_{it})$			
	(1)	(2)	(3)	(4)
60–40 days pre	0.0729	0.0513	0.0628	0.0070
$Subwayn_{it}\times W\left(\mathrm{od}-60\leq t<\mathrm{od}-40\right)$	(0.0539)	(0.0389)	(0.0399)	(0.0640)
40–20 days pre	−0.0653	−0.0569	−0.0456	−0.0700
$Subwayn_{it}\times W\left(\mathrm{od}-40\leq t<\mathrm{od}-20\right)$	(0.0555)	(0.0453)	(0.0473)	(0.0730)
0–20 days post	−0.1155 ***	−0.0879 **	−0.0766 *	−0.0731
$Subwayn_{it}\times W\left(\mathrm{od}<t\leq \mathrm{od}+20\right)$	(0.0427)	(0.0407)	(0.0425)	(0.0643)
20–40 days post	−0.0998 **	−0.1245 ***	−0.1132 **	−0.0665
$Subwayn_{it}\times W\left(\mathrm{od}+20<t\leq \mathrm{od}+40\right)$	(0.0450)	(0.0427)	(0.0445)	(0.0586)
40–60 days post	−0.1689 ***	−0.1465 ***	−0.1354 ***	−0.0811
$Subwayn_{it}\times W\left(\mathrm{od}+40<t\leq \mathrm{od}+60\right)$	(0.0389)	(0.0397)	(0.0419)	(0.0550)
Time Window (days)	±60	±60	±60	±60
Weather Controls	Y	Y	Y	Y
Time FE	N	Y	Y	Y
Monitor FE	N	N	Y	Y
Monitor FE × Trend	N	N	N	Y
Observations	2178	2178	2178	2178
R^2^	0.2246	0.2699	0.3028	0.3462

Notes: Robust standard errors in parentheses clustered at the day level, *** *p* < 0.01, ** *p* < 0.05, * *p* < 0.1. FE indicates fixed effect. od represents opening dates.

**Table 3:** The effect of subway network density on air pollutants: Ordinary Least Squares (OLS).

Dependent Variable:	$ln(AQI_{it})\;$			$ln(PM_{2.5}{}_{it})\;$	$ln(PM_{10}{}_{it})\;$	$ln(NO_{2}{}_{it})$	$ln(CO_{it})$	$ln(O_{3}{}_{it})$	$ln(SO_{2}{}_{it})$
	(1)	(2)	(3)	(4)	(5)	(6)	(7)	(8)	(9)
$Subway_{d}ensityst_{it}$	0.0105 ***	−0.0687 ***	−0.0705 ***	−0.1982 ***	−0.0848 ***	−0.1781 ***	−0.0044	0.2380 ***	−0.0694 **
	(0.0011)	(0.0100)	(0.0221)	(0.0301)	(0.0257)	(0.0218)	(0.0238)	(0.0226)	(0.0289)
rain	−0.0140	−0.0136	−0.0156	0.0857 ***	0.0132	0.0968 ***	0.1266 ***	−0.1375 ***	−0.0229
	(0.0220)	(0.0220)	(0.0218)	(0.0305)	(0.0266)	(0.0183)	(0.0166)	(0.0276)	(0.0221)
toptem	0.0617 ***	0.0617 ***	0.0616 ***	0.0634 ***	0.0757 ***	0.0414 ***	0.0332 ***	0.0432 ***	0.0634 ***
	(0.0035)	(0.0035)	(0.0035)	(0.0051)	(0.0045)	(0.0030)	(0.0029)	(0.0046)	(0.0037)
minitem	−0.0630 ***	−0.0631 ***	−0.0625 ***	−0.0724 ***	−0.0779 ***	−0.0481 ***	−0.0329 ***	−0.0150 ***	−0.0609 ***
	(0.0039)	(0.0039)	(0.0039)	(0.0057)	(0.0050)	(0.0032)	(0.0032)	(0.0051)	(0.0040)
windd	−0.0125	−0.0123	−0.0099	−0.0262	−0.0003	−0.0312 **	−0.0423 ***	0.0712 ***	0.0097
	(0.0182)	(0.0182)	(0.0182)	(0.0262)	(0.0220)	(0.0154)	(0.0141)	(0.0210)	(0.0179)
winds	−0.2032 ***	−0.2032 ***	−0.2037 ***	−0.2916 ***	−0.2061 ***	−0.2321 ***	−0.1664 ***	0.0569 ***	−0.1060 ***
	(0.0167)	(0.0167)	(0.0165)	(0.0235)	(0.0206)	(0.0126)	(0.0146)	(0.0199)	(0.0151)
Time FE	Y	Y	Y	Y	Y	Y	Y	Y	Y
Monitor FE	N	Y	Y	Y	Y	Y	Y	Y	Y
Monitor FE × Trend	N	N	Y	Y	Y	Y	Y	Y	Y
Observations	15,070	15,070	15,070	15,066	15,043	15,071	15,066	15,069	15,071
R^2^	0.4405	0.4476	0.4510	0.4296	0.4511	0.3713	0.3018	0.4854	0.3958

Notes: Robust standard errors in parentheses clustered at the day level, *** *p* < 0.01, ** *p* < 0.05. The unit of observation is monitor-day. All pollutants are expressed in micrograms per cubic meter and are all logarithmically transformed.

**Table 4:** Estimation results based on difference-in-differences analysis (DID).

	$\mathrm{Dependent}\;\mathrm{Variable}:\;ln(AQI_{it})$						
	(1)	(2)	(3)	(4)	(5)	(6)	(7)
$Subwayn_{it}\times WPost_{t}$	−0.0736 **	−0.1177 ***	−0.1081 ***	−0.0965 ***	−0.0393 *		−0.0396 *
	(0.0290)	(0.0220)	(0.0196)	(0.0229)	(0.0229)		(0.0238)
$Subwayn4_{it}\times WPost_{t}$						−0.1181 ***	
						(0.0231)	
rain		−0.0557	−0.0683	−0.0683	−0.0761	−0.0544	−0.0823
		(0.0542)	(0.0549)	(0.0550)	(0.0524)	(0.0456)	(0.0521)
toptem		0.0450 ***	0.0505 ***	0.0504 ***	0.0581 ***	0.0655 ***	0.0598 ***
		(0.0075)	(0.0073)	(0.0073)	(0.0072)	(0.0062)	(0.0072)
minitem		−0.0413 ***	−0.0348 ***	−0.0348 ***	−0.0285 ***	−0.0517 ***	−0.0304 ***
		(0.0086)	(0.0089)	(0.0089)	(0.0097)	(0.0077)	(0.0097)
windd		−0.0092	0.0091	0.0089	0.0204	−0.0291	0.0226
		(0.0424)	(0.0419)	(0.0420)	(0.0412)	(0.0336)	(0.0413)
winds		−0.1779 ***	−0.1614 ***	−0.1618 ***	−0.1423 ***	−0.1989 ***	−0.1627 ***
		(0.0459)	(0.0467)	(0.0468)	(0.0457)	(0.0354)	(0.0458)
Time Window (days)	od ± 60	od ± 60	od ± 60	od ± 60	od ± 60	od ± 60	od ± 60
Year FE	N	N	Y	Y	Y	Y	Y
Season FE	N	N	Y	Y	Y	Y	Y
Weekend	N	N	Y	Y	Y	Y	Y
Holiday	N	N	Y	Y	Y	Y	Y
Monitor FE	N	N	N	Y	Y	Y	Y
Monitor FE × Trend	N	N	N	N	Y	Y	Y
Hour FE	N	N	N	N	N	N	Y
Observations	2178	2178	2178	2178	2178	4172	49,943
R^2^	0.0043	0.2193	0.2663	0.2994	0.3447	0.3745	0.2496

Notes: The unit of observation is monitor-day. Robust standard errors in parentheses clustered at the day level. Significance levels: *** *p* < 0.01, ** *p* < 0.05, * *p* < 0.1.

**Table 5:** DID with varying time windows.

	$\mathrm{Dependent}\;\mathrm{Variable}:\;ln(AQI_{it})$					
	(1)	(2)	(3)	(4)	(5)	(6)
$Subwayn_{it}\times WPost_{t}$	0.0951 **	0.0795 **	0.0168	−0.0353	−0.0250	−0.0319
	(0.0388)	(0.0383)	(0.0326)	(0.0286)	(0.0256)	(0.0215)
Time Window (days)	od ± 10	od ± 20	od ± 30	od ± 40	od ± 50	od ± 70
	(7)	(8)	(9)	(10)	(11)	(12)
$Subwayn_{it}\times WPost_{t}$	−0.0590 ***	−0.0650 ***	−0.0633 ***	−0.0685 ***	−0.0553 ***	−0.0534 ***
	(0.0206)	(0.0205)	(0.0192)	(0.0199)	(0.0189)	(0.0179)
Time Window (days)	od ± 80	od ± 90	od ± 100	od ± 110	od ± 120	od ± 130
	(13)	(14)	(15)	(16)	(17)	(18)
$Subwayn_{it}\times WPost_{t}$	−0.0565 ***	−0.0574 ***	−0.0533 ***	−0.0411 ***	−0.0348**	−0.0662 ***
	(0.0173)	(0.0166)	(0.0159)	(0.0156)	(0.0149)	(0.0191)
Time Window (days)	od ± 140	od ± 150	od ± 160	od ± 170	od ± 180	od ± 60

Notes: Robust standard errors in parentheses clustered at the day level, *** *p* < 0.01, ** *p* < 0.05. All columns include the following controls: the daily weather fixed effect, time fixed effects, and dummies for monitors and the interactions with the time trend. od represents opening dates.

**Table 6:** DID-based estimation of different time intervals and types of air pollutants.

Service Time (6:00–23:00)	(1)	(2)	(3)	(4)	(5)	(6)
Dependent Variable:	$ln(PM_{2.5}{}_{it})$	$ln(PM_{10}{}_{it})$	$ln(CO_{it})$	$ln(NO_{2}{}_{it})$	$ln(O_{3}{}_{it})$	$ln(SO_{2}{}_{it})$
$Subwayn_{it}\times WPost_{t}$	−0.0685 **	−0.0390	−0.1080 ***	0.0531 **	0.0531 *	−0.0264
	(0.0329)	(0.0270)	(0.0269)	(0.0243)	(0.0307)	(0.0336)
Time window	od ± 60	od ± 60	od ± 60	od ± 60	od ± 60	od ± 60
Observations	37,114	36,805	37,210	37,289	37,275	37,292
R-squared	0.2437	0.3125	0.2389	0.3432	0.5378	0.3158
Service time (7:00–9:00/17:00–19:00)	(7)	(8)	(9)	(10)	(11)	(12)
$Subwayn_{it}\times WPost_{t}$	−0.0567 *	−0.0308	−0.1072 ***	0.0564 **	0.0655 **	−0.0267
	(0.0339)	(0.0279)	(0.0265)	(0.0248)	(0.0313)	(0.0343)
Time window	od ± 60	od ± 60	od ± 60	od ± 60	od ± 60	od ± 60
Observations	12,526	12,421	12,561	12,586	12,579	12,586
R^2^	0.2381	0.2999	0.2585	0.3603	0.5337	0.2957
Out of service (1:00–5:00)	(13)	(14)	(15)	(16)	(17)	(18)
$Subwayn_{it}\times WPost_{t}$	−0.0496	−0.0273	−0.1100 ***	0.0398	0.1012 **	−0.0152
	(0.0384)	(0.0330)	(0.0285)	(0.0310)	(0.0422)	(0.0380)
Time window	od ± 60	od ± 60	od ± 60	od ± 60	od ± 60	od ± 60
Observations	10,506	10,382	10,528	10,545	10,535	10,543
R^2^	0.1834	0.2426	0.1990	0.3256	0.5420	0.2269
Time range (0:00–24:00)	(19)	(20)	(21)	(22)	(23)	(24)
$Subwayn_{it}\times WPost_{t}$	−0.0636**	−0.0390	−0.1092 ***	0.0496 **	0.0621 *	−0.0235
	(0.0317)	(0.0270)	(0.0261)	(0.0239)	(0.0320)	(0.0332)
Time window	od ± 60	od ± 60	od ± 60	od ± 60	od ± 60	od ± 60
Observations	49,709	36,805	49,830	49,933	49,908	49,934
R^2^	0.2234	0.3125	0.2249	0.3319	0.5300	0.2862

Notes: Robust standard errors in parentheses clustered at the day level, *** *p* < 0.01, ** *p* < 0.05, * *p* < 0.1. All columns control for the daily weather fixed effect, time fixed effects (year, holiday, weekend, season, hour), and dummies for air pollution monitoring stations and the interactions with the time trend. The dependent variable is the pollution concentration of each pollutant for a specific hour of a day.

## Data Availability

The data presented in this study are available on request from the corresponding author.
